# Interaction of Pexiganan (MSI-78)-Derived Analogues Reduces Inflammation and TLR4-Mediated Cytokine Secretion: A Comparative Study

**DOI:** 10.1021/acsomega.3c00850

**Published:** 2023-05-12

**Authors:** Hadar Cohen, Naiem Ahmad Wani, Daniel Ben Hur, Ludovico Migliolo, Marlon H. Cardoso, Ziv Porat, Eyal Shimoni, Octavio Luiz Franco, Yechiel Shai

**Affiliations:** † Department of Biomolecular Sciences, The Weizmann Institute of Science, Rehovot 76100, Israel; ‡ Departamento de Engenharia Sanitária e Ambiental, Universidade Católica Dom Bosco, Campo Grande 79117-900, Brazil; § The Department of Life Sciences Core Facilities, The Weizmann Institute of Science, Rehovot 76100, Israel; ∥ Department of Chemical Research Support, The Weizmann Institute of Science, Rehovot 76100, Israel; ⊥ S-Inova, Programa de Pós-Graduação em Biotecnologia, Universidade Católica Dom Bosco, Campo Grande 79117900, MS, Brazil; # Centro de Análises Proteômicas e Bioquímicas, Pós-Graduação em Ciências Genômicas e Biotecnologia, Universidade Católica de Brasília, Brasília 70790160, DF, Brazil; ∇ Instituto de Biociências (INBIO), Universidade Federal de Mato Grosso do Sul, Cidade Universitária, Campo Grande 79070900, Mato Grosso do Sul, Brazil

## Abstract

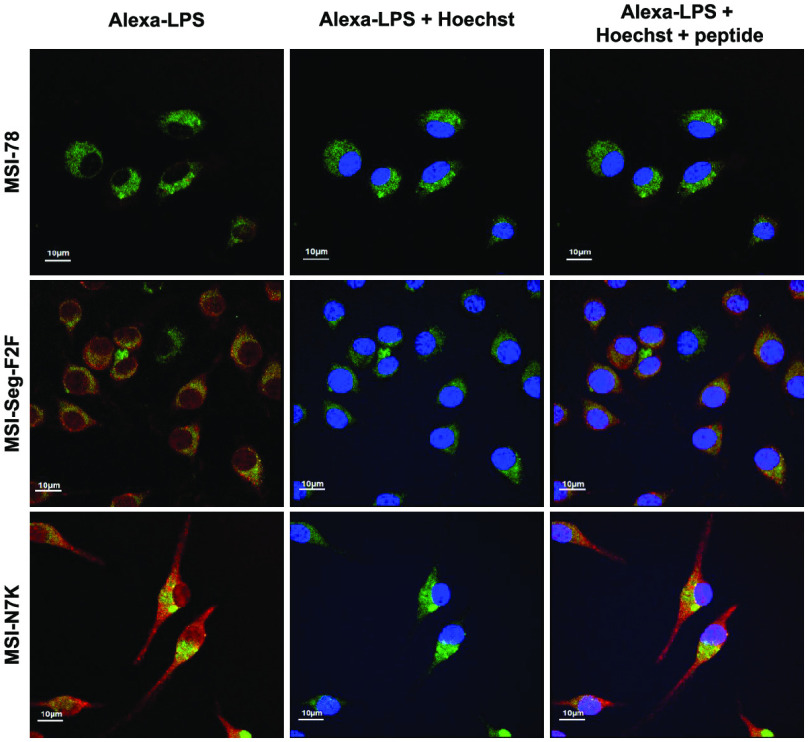

Antibiotic-resistant bacterial infections have increased the prevalence of sepsis and septic shock mortality worldwide and have become a global concern. Antimicrobial peptides (AMPs) show remarkable properties for developing new antimicrobial agents and host response modulatory therapies. A new series of AMPs derived from pexiganan (MSI-78) were synthesized. The positively charged amino acids were segregated at their N- and C-termini, and the rest of the amino acids created a hydrophobic core surrounded by positive charges and were modified to simulate the lipopolysaccharide (LPS). The peptides were investigated for their antimicrobial activity and LPS-induced cytokine release inhibition profile. Various biochemical and biophysical methods were used, including attenuated total reflection Fourier transform infrared (ATR-FTIR) spectroscopy, microscale thermophoresis (MST), and electron microscopy. Two new AMPs, MSI-Seg-F2F and MSI-N7K, preserved their neutralizing endotoxin activity while reducing toxicity and hemolytic activity. Combining all of these properties makes the designed peptides potential candidates to eradicate bacterial infection and detoxify LPS, which might be useful for sepsis treatment.

## Introduction

Severe inflammatory reactions that result in sepsis and septic shock can be induced by bacterial infections and their associated endotoxins, such as lipopolysaccharide (LPS) from Gram-negative bacteria.^1^ With the rise of multidrug-resistant Gram-negative bacteria and the ineffectiveness of current treatment methods, there is a pressing need for the development of antiendotoxin agents.^2^ Therefore, it is crucial to identify alternative drugs that can efficiently control infections and curb inflammatory responses. Lipopolysaccharide (endotoxin) is the major cell wall surface component of Gram-negative bacteria, which provides a physical permeability barrier that protects the bacteria from antibacterial agents.^3−8^ The molecule contains negatively charged carboxyl and phosphate groups and consists of a lipid moiety (lipid A) that is hydrophobic, a short oligosaccharide (R-core), and an outer region composed of polymeric carbohydrates (O-antigen).^9^ Due to its heightened peptide charge and hydrophobicity, LPS is capable of binding to cationic and amphiphilic peptides by means of the combined effects of its lipid A and carbohydrate region.^10^

The ability of the peptides to block and neutralize pathogen-associated molecular patterns (PAMPs) has been well documented for LPS produced by Gram-negative bacteria. LPS is considered one of the most immunogenic PAMPs. Once released, they are mainly recognized by Toll-like receptor 4 (TLR4), which triggers an inflammatory response that controls the infection and clears the pathogen. LPS recognition promotes TLR4-induced inflammation as part of the host response, leading to the activation of phagocytes, monocytes, and dendritic cells via TLR4, which stimulates proinflammatory cytokine secretion such as TNFα, IL-6, IL-12, and IL-1β.^11,12^ An unbalanced immune response and the presence of LPS in circulation leads to increased secretion of the inflammatory cytokines, leading to the establishment of sepsis and septic shock.^12,13^ For that reason, new agents that reduce circulating LPS are highly needed. Antimicrobial peptides (AMPs), known for their antimicrobial and antibiofilm properties, are essential in innate immunity and also have anti-inflammatory and immune-modulating effects. These peptides may offer a viable solution in the treatment of sepsis.^6,14−21^

Pexiganan (MSI-78) is an antimicrobial peptide composed of 22 amino acids derived from magainin,^22−24^ with previously demonstrated antimicrobial and anti-inflammatory effects.^25^ The structure–function relationship has shown how peptide length, charge, hydrophobicity, and secondary structure affect antiendotoxic effects.^26,27^ To facilitate mechanistic interpretation using biophysical and biochemical methods, the studies include peptides of identical length and composition, in which the native sequence of MSI-78 has been segregated by sorting its charged amino acid lysine toward both the N- and C-termini. This sequence arrangement with altered structures was to create a hydrophobic core, which was modified by charge segregation (MSI-Seg), replacement of amino acids to reduce hydrophobicity (MSI-SegF2G), chirality (MSI-SegF2F), charge clustering (MSI-N7K), and scrambling (MSI-Seg-Scr) of amino acid residues. These peptides were investigated for antibacterial activity against a panel of two Gram-negative bacteria (*Salmonella Typhi* ATCC 14028s and *Escherichia coli* ATCC 35218) and two Gram-positive bacteria (*Staphylococcus aureus* ATCC P8538 and *Lactobacilli* ATCC 25258). Interestingly, all of the new peptides retained some antibacterial activity. The peptides were investigated for their ability to inhibit LPS-induced cytokine release, their direct effect on LPS micelles, and their behavior on macrophages. This might allow the binding of peptides to the LPS via their hydrophobic moieties and between the positive charges of the peptides and the phosphate groups of LPS.

## Results

### Peptide’s Designations, Sequences, and Relative Hydrophobicity

**Figure 1:**
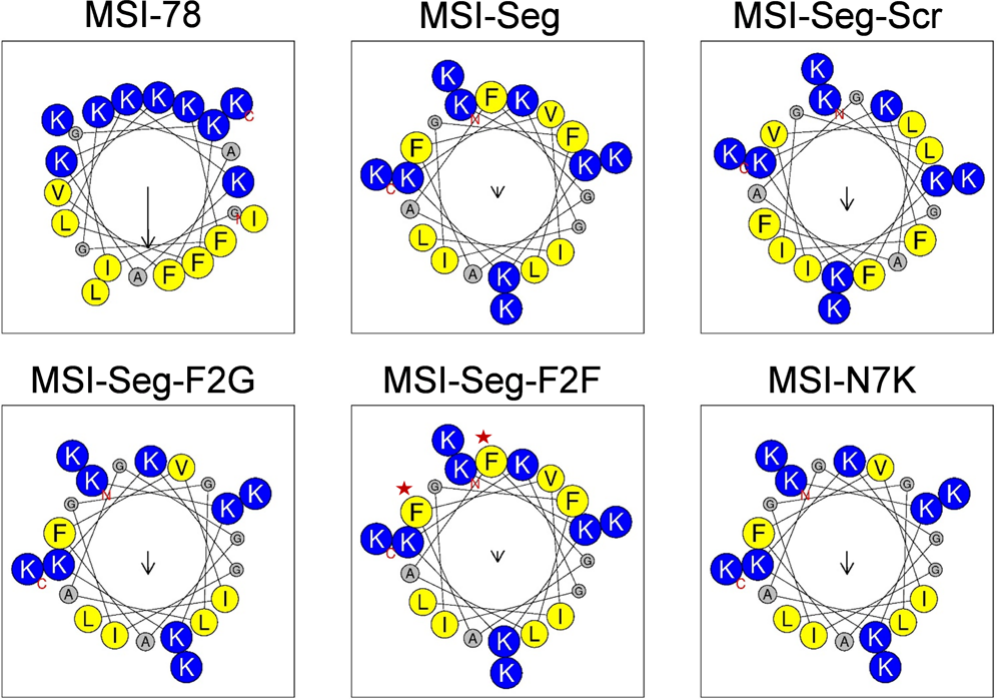
Helical wheel projections of all MSI-78 and its analogues. Positively charged residues are shown in blue, hydrophobic residues are shown in yellow, and Gly and Ala are shown in gray. The arrows indicated the orientation of hydrophobic moment. The stars on MSI-Seg-F2F are an indication of the d-enantiomers.

Five new AMPs were synthesized from the well-known AMP pexiganan (MSI-78) based on the general character of the LPS molecule, which includes a hydrophobic region (lipid A moiety) and charge (core oligosaccharide with charged phosphate groups).^5,28^ We performed charge clustering and multiple amino acid changes on the hydrophobic and hydrophilic faces of the helical structure (Figure 1). All of these peptides have at least one charged residue within the peptide sequence, which prevents complete segregation of charged residues to one end. Therefore, some of the peptides still have high amphipathicity, as determined by hydrophobic moment (Table 1). The peptides were designed as follows: segregation lysines in the N- and C-termini (MSI-Seg), scrambling of the core hydrophobic region (MSI-Seg-Scr), changing the chirality of the hydrophobic amino acid region by replacing two Phe with their D-optical isomers (MSI-Seg-F2F), reducing the relative hydrophobicity of the core region by replacing two Phe with two Gly (MSI-Seg-F2G), and moving most of the charged amino acids to the N-terminal (MSI-N7K). The HPLC retention time is known to reflect the relative hydrophobicity of peptides. The relative hydrophobicity of MSI-Seg, MSI-Seg-Scr, and MSI-Seg-F2F is similar to that of the MSI-WT. The relatively low hydrophobicity of MSI-Seg-F2G is due to the replacement of two Phe with Gly. In comparison, due to the exposed hydrophobic C-terminal of MSI-N7K, the hydrophobicity is relatively high (Table 1).

**Table 1:**
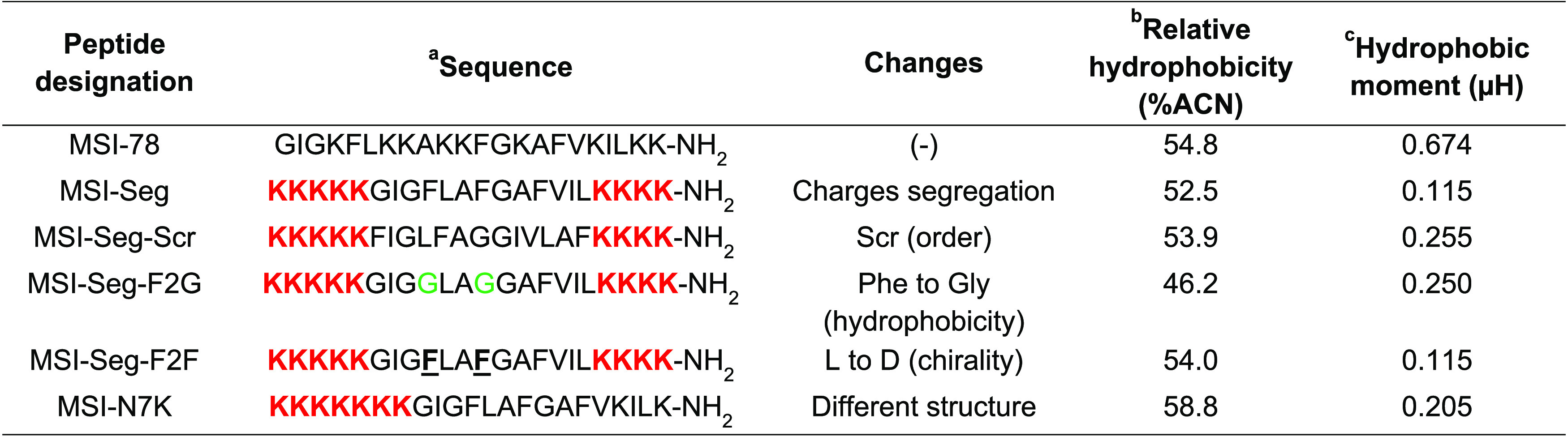
Designation, Sequence, and Relative Hydrophobicity of MSI-Derived Peptides

a Underlined and bolded amino acids are d-enantiomers. Green-labeled amino acids represent the change in sequence compared to the MSI-Seg peptide. Lysines are labeled in red. All peptides are amidated in their C-terminus.

b Peptides were evaluated in a C18 reverse-phase analytic column for 40 min, using a linear gradient from 10 to 90% acetonitrile in water, containing 0.1% TFA.

c Hydrophobic moment (μH) of AMPs was calculated using HeliQuest (http://heliquest.ipmc.cnrs.fr).

### Antimicrobial Activity and Toxicity of MSI-Derived Peptides

The peptide’s ability to inhibit the growth of bacteria was assessed by testing them against both Gram-negative bacteria, such as *Salmonella Typhi* and *Escherichia coli*, and Gram-positive bacteria, including *Staphylococcus aureus* and *Lactobacillus*. (Table 2). The data revealed that MSI analogue peptides preserved some activity against all of the bacterial strains, except MSI-Seg-F2G, which was not active even at the highest tested concentration, as shown from the minimal inhibitory concentration (MIC) experiments (Table 1). Further, the peptide’s toxicity was examined on macrophages (RAW 264.7 cells) and erythrocytes (RBCs). The data demonstrated that all of the peptides were not toxic against RAW 264.7 cells even at the highest concentration tested in contrast to MSI-78 and MSI-N7K, which were toxic at 40 and 60 μM, respectively. Moreover, hemolysis experiments showed that MSI-78 and MSI-Seg were slightly hemolytic at 100 μM, the highest concentration which has been tested (Table 2).

**Table 2:** Antimicrobial Activity and Toxicity of MSI-Derived Peptides

peptide designation	*S. Typhi*^a^ ATCC 14028s	*E. coli*^a^ ATCC 35218	*S. aureus*^a^ ATCC P8538	*Lactobacilli*^a^ ATCC 25258	LD_50_^b^ (RAW 264.7)	% hemolysis^c^ (100 μM)
MSI-78	12.5	3.12	6.25	1.56	43.1	15.2 ± 1.1
MSI-Seg	25	12.5	12.5	>50	>100	16.6 ± 4.2
MSI-Seg-Scr	25	12.5	12.5	12.5	>100	3.9 ± 1.4
MSI-Seg-F2G	>50	>50	25	>50	>100	1.2 ± 30.2
MSI-Seg-F2F	25	12.5	12.5	12.5	>100	8.1 ± 3.2
MSI-N7K	25	25	25	25	60.9	10 ± 5.6

a The antimicrobial activity is represented by MIC in μM.

b The median lethal dose (LD_50_) is represented in μM.

c The data represent the mean ± standard error of the means (S.E.Ms) from three biological repeats.

### Neutralization of LPS-Mediated Cytokine Secretion by MSI-Derived Peptides

**Figure 2:**
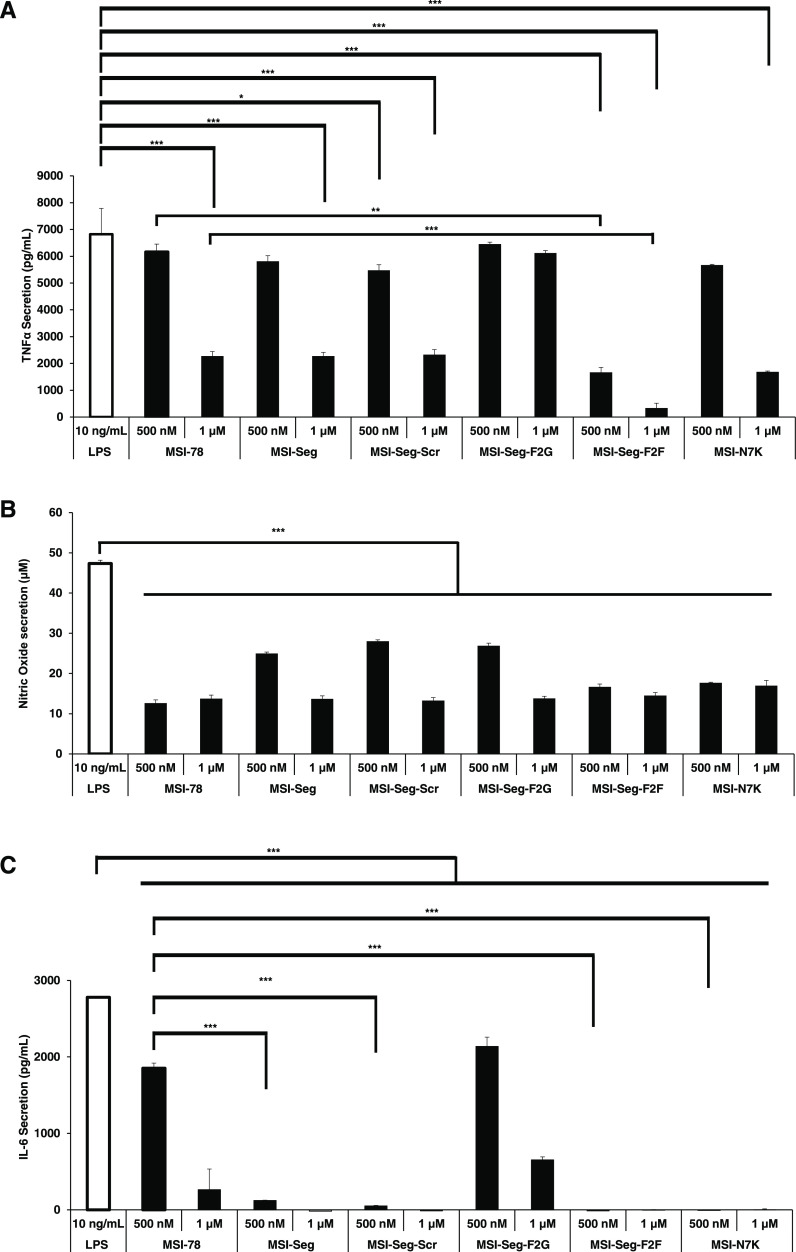
Effect of peptides on cytokine secretion: (A) TNF-α secretion, (B) NO secretion, and (C) IL-6 secretion. RAW 264.7 cells were stimulated with LPS (10 ng mL^–1^) in the absence or presence of different peptides at 500 nM and 1 μM. 4 h for TNFα or 24 h for IL-6 and NO; supernatant of the cell’s medium was evaluated for their cytokine concentration by Griess and enzyme-linked immunosorbent assay (ELISA). The results represent a percentage normalized to cells stimulated with LPS. Untreated cells served as control. Statistical analyses were performed via one-way ANOVA *P ≤ 0.05, **P ≤ 0.01, and ***P ≤ 0.001.

The neutralization of inflammatory cell activation by LPS is achieved through the interaction of peptides with LPS, which causes biophysical changes and results in an LPS structure that is biologically less active. LPS rapidly activates the immune system by its main receptor, TLR4. TLR4 activation leads to the secretion of proinflammatory mediators such as cytokines and chemokines, including TNF-α, IL-6, IL-12, IL-1β, and NO. These mediators play an immunoregulatory role in the induction and resolution of inflammation, which may lead to septic shock.^29,30^ Here, we examined the peptide’s ability to inhibit LPS-mediated inflammatory response on RAW 264.7 macrophages that express all TLR family using LPS (10 ng mL^–1^). The ability of the peptides to detoxify LPS was monitored by the secretion of TNF-α, NO, and IL-6. The results revealed that peptides MSI-Seg-F2F, followed by MSI-N7K, were potent inhibitors. The other peptides, including MSI-78, were slightly less active, and the peptides MSI-Seg-F2G were not active at all tested concentrations. Further, to detect the amount of NO in the medium with and without the presence of the peptides using the Griess reagent revealed a similar trend in the activities of the peptides (Figure 2A–C). Furthermore, the peptides were tested toward Pam3CSK4 (100 ng mL^–1^), an analogue of LTA, that highly activates TLR2, which also promotes TNFα secretion via the NFkB pathway. From this experiment, it was demonstrated that all of the peptides significantly reduced/neutralized Pam3CSK4 at 1 μM (Figure S2) The disparity between LPS and LTA lies predominantly in their respective headgroups, which could potentially impact the binding of peptides to these molecules. Peptides that neutralize LPS may do so by engaging in robust interactions with the negatively charged LPS molecule, causing subsequent physicochemical alterations to its structure.

### Secondary Structure of Peptides in LPC and LPS Suspension Determined by Attenuated Total Reflection Fourier Transform Infrared (ATR-FTIR) Spectroscopy

**Figure 3:**
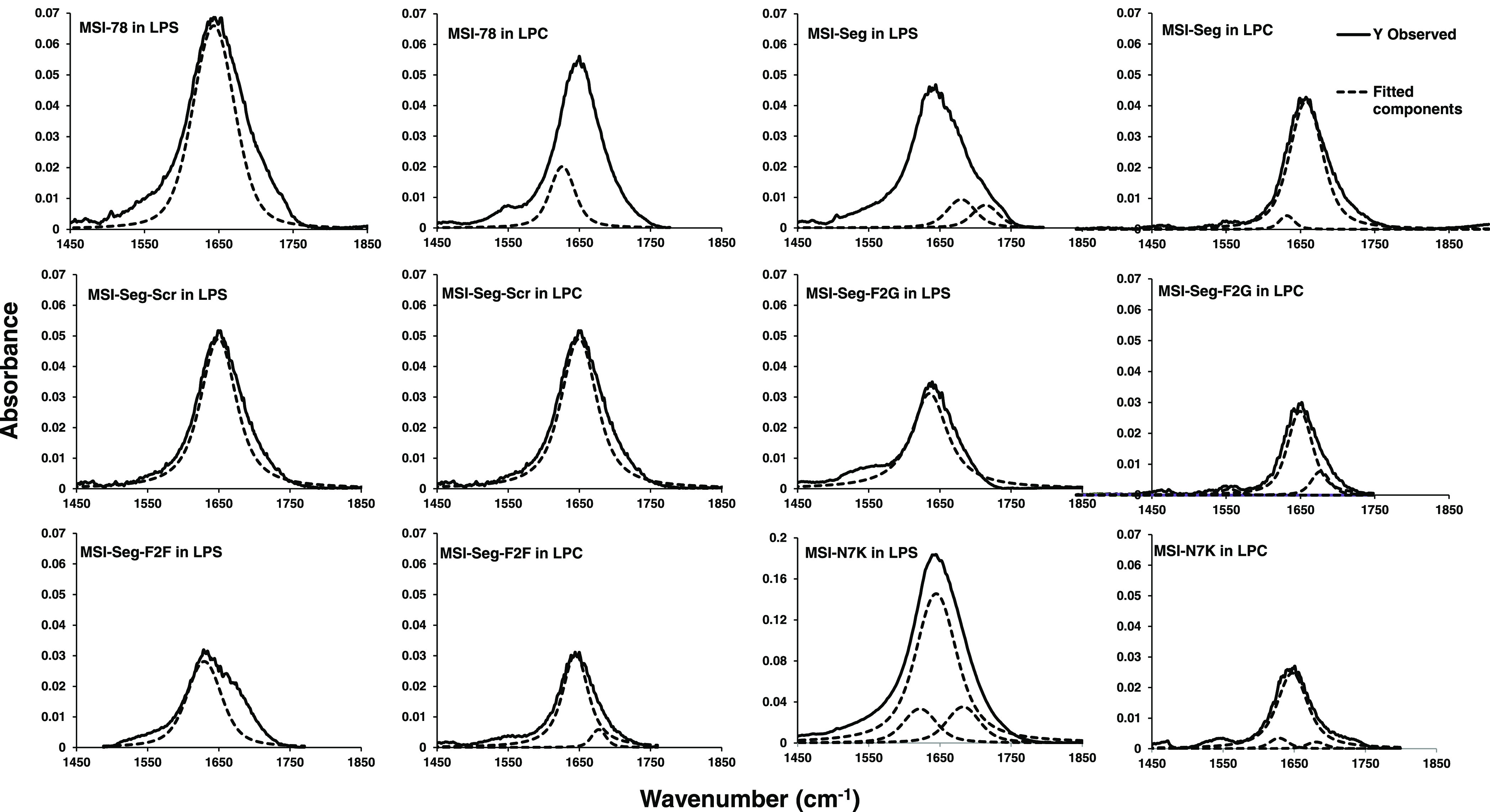
ATR-FTIR spectra for the amide(I) range of MSI-78 and its analogues in LPS and LPC. Solid lines represent the experimental FTIR spectra.

ATR-FTIR spectroscopy was used to get information on the secondary structure of peptides from the analysis of the strong amide I band. We investigated the structural behavior of MSI-78 and their analogues when interacting with the LPS and lysophosphatidylcholine (LPC), which mimics the mammalian membrane. Figure 3 shows the ATR-FTIR spectra of MSI-78 and its analogue peptides interacting with LPS and LPC. The results revealed that all of the peptides have a secondary structure in the negatively charged LPS compared to the LPC environment (Table 3). MSI-78 adopted an α-helical structure in LPC and LPS, whereas MSI-Seg-F2F exhibited a predominantly β-sheet structure. Nevertheless, MSI-Seg and MSI-Seg-Scr adopt α-helical structures in LPC. Moreover, MSI-Seg-F2G shows a random coil in LPC. In comparison to LPC, the segregated peptides exhibited the β-sheet structures except for MSI-N7K, which displayed a random coil structure in both LPS and LPC.

**Table 3:** Secondary Structure Content Derived from ATR-FTIR Spectra of MSI-78 and Its Analogues Interacting with LPC and LPS^a^

	LPC				LPS			
peptide	α helix	β structure	random coil	other	α helix	β structure	random coil	other
MSI-78	**61**	21.58		17.42	74.9			25.1
MSI-Seg	**87.12**	4.2		8.68		**83.9**		16.14
MSI-Seg-Scr	**86.8**			13.16		**94.25**		5.75
MSI-Seg-F2G		22.5	**70.3**	7.2	12.51	**75.77**		11.79
MSI-Seg-F2F		**88.14**		11.86	17.39	**68.42**		14.21
MSI-N7K		10.64	**81.46**	7.86		13.11	**72.2**	14.89

a All peptides were examined by using FTIR analysis to determine their secondary structure both in LPC and in LPS. The data are presented as percentage.

MSI-78 is amphipathic and helical, by charge clustering, i.e., when all of the lysines are moved to the N-terminus in the peptide MSI-N7K, the peptide loses its amphipathicity as well as structure, while segregated peptides retained the amphipathicity and adopted the β-sheet structures, which is known to neutralize the LPS.^31^ Among the segregated peptides, MSI-Seg and MSI-Seg-Scr were active against bacterial strains tested but did not neutralize the LPS. The MSI-N7K amino acid sequence suggests that it has a low hydrophobic moment of 0.068, indicating that it is unlikely to be drawn into lipid bilayers. However, it is more effective at neutralizing LPS, possibly due to charge clustering. On the other hand, MSI-F2F achieves this through chirality changes.

### Analysis of the Interaction between MSI-Derived Peptides and LPS by Using Microscale Thermophoresis

**Figure 4:**
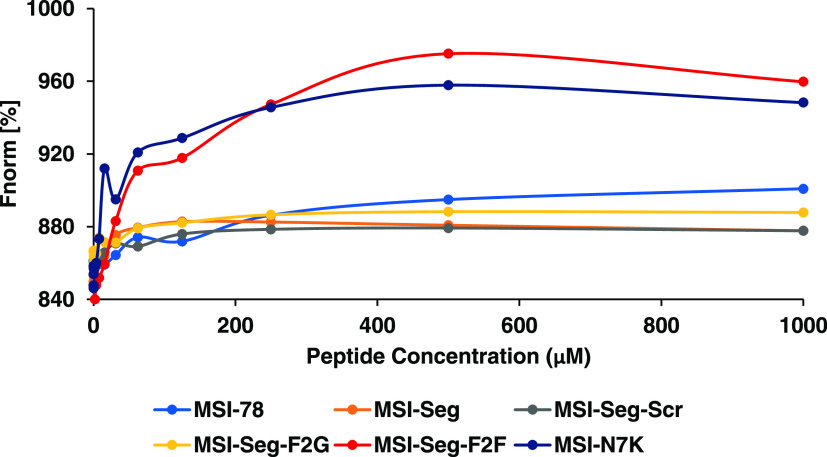
Microscale thermophoresis assay quantifying interactions between MSI-derived peptides and LPS-FITC. MST analysis was performed in the concentration range of 1000–0.01 μM. *K*_d_ constants of MSI-78 = 16 μM, MSI-Seg = 16.8 μM, MSI-Seg-Scr = 38.5 μM, MSI-Seg-F2G = 40 μM, MSI-Seg-F2F = 71 μM, and MSI-N7K = 25.5 μM were determined.

Microscale thermophoresis (MST) is a powerful technique to quantify biomolecular interactions using a temperature gradient, allowing accurate analysis of the binding events. Therefore, we checked and calculated the interaction between LPS fluorescein isothiocyanate (FITC) labeled with MSI-derived peptides. All of the peptides have the ability to bind to LPS at micromolar concentrations, however, MSI-Seg-F2F and MSI-N7K displayed higher values compared to other peptides (Figure 4).

### Molecular Modeling of MSI-Derived Peptides Bound to the LPS Molecule

MSI-derived peptides had their tridimensional structures predicted by ab initio approaches, followed by a validation of the procedures. All theoretical models presented good folding quality on ProSA,^32^ with *z*-scores ranging from 0.08 to −1.48, with referenence to NMR structures deposited on the Protein Data Bank (PDB). Moreover, the calculated average score for the dihedral angles from all models showed values > −0.5, indicating a reliable structure. Additionally, >90% of the residues of the MSI-derived peptide could be assigned in the most favored regions in the Ramachandran plot except for MSI-Seg (88.2%).

**Figure 5:**
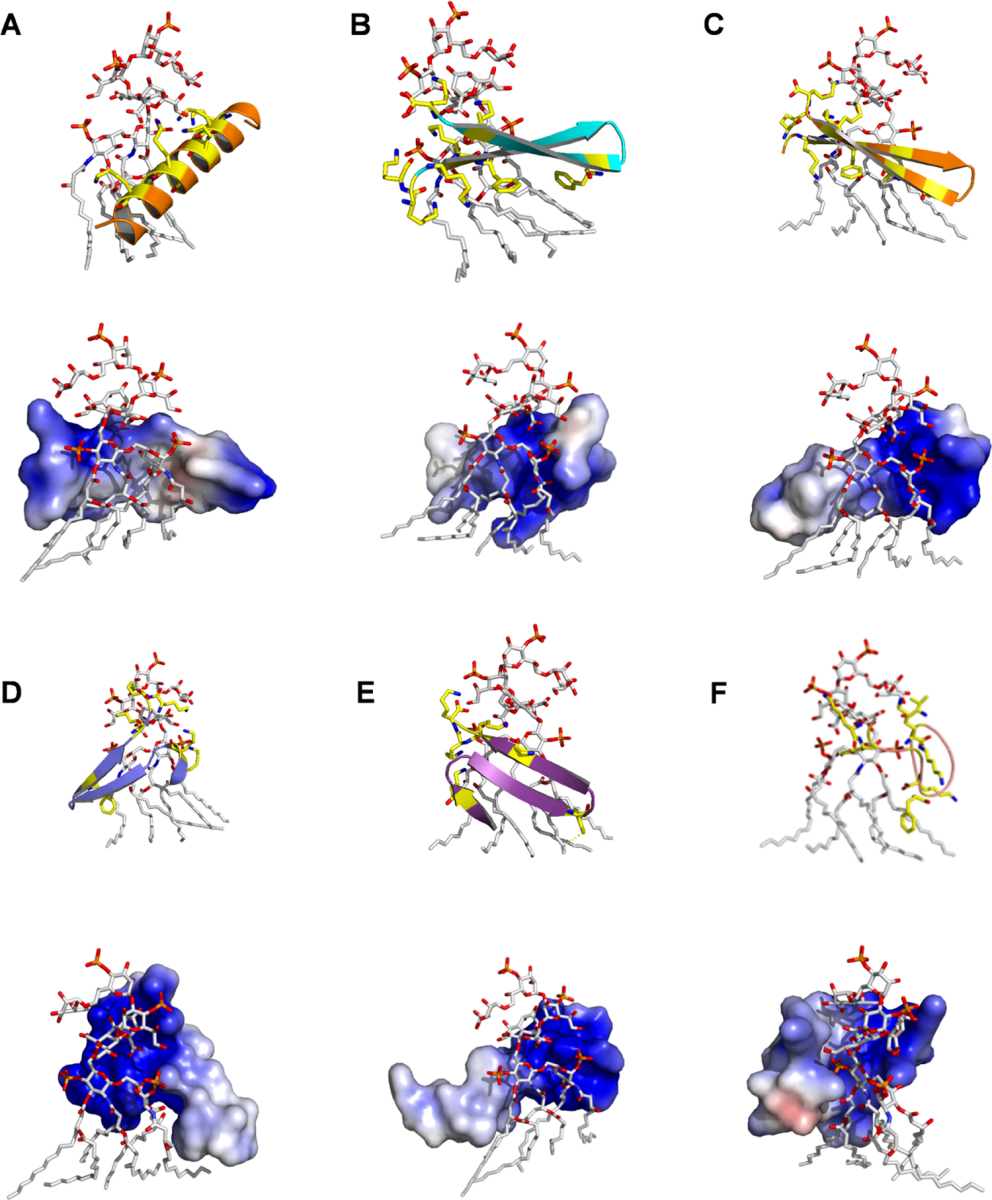
Molecular docking stimulation of MSI-derived peptides toward LPS, calculated affinities, and potential conformation. (A) MSI-78, (B) MSI-Seg, (C) MSI-Seg-Scr, (D) MSI-Seg-F2G, (E) MSI-Seg-F2F, and (F) MSI-N7K. Each figure consists of a secondary structure and an electrostatic surface potential below from (−10.9)*kT*/*e* (red) to (+10.1)*kT*/*e* (blue). Additionally, the hydrophobic patches are present in white.

The purpose of programming molecular docking simulations was to gain a deeper understanding of how peptides interact with bacterial LPS. Therefore, we calculated the affinity of the different peptides and LPS complexes while predicting their atomic interactions. The affinities of all complexes are summarized in Table S1, ranging from −4.1 to −4.4 kcal mol^–1^, the best affinities (−4.4 kcal mol^–1^) observed for MSI-Seg-F2F and MSI-N7K. As observed in our experimental data FTIR, MSI-78 and all β-sheet MSI-derived peptides preserved their structural conformations during docking simulations, presenting only a few variations on the side chains (Figure 5A–E). At the same time, MSI-N7K adopted random coil arrangement (Figure 5F). Among the atomic interactions, hydrogen and saline bonds, the hydrophobic interactions could be predicted with distances ranging from 2.8 to 3.6 Å. As expected, for all complexes, hydrophobic interactions were mainly established between the side chains of hydrophobic residues from the peptide and carbon atoms from the acyl chains from the LPS molecule. In addition, negatively charged phosphate groups on the upper portion of the LPS seem to play an essential role in peptides binding through electrostatic interactions involving the lysine amine (NZ) atoms of lysine residues. Furthermore, hydrogen bonds are also distributed along the glucosamine and 3-deoxy-d-manno-2-octulosonic acid groups from the LPS. These properties could also be observed based on the calculated surface electrostatic potential for each peptide after the docking simulation, where cationic and polar regions of all MSI-derived peptides are clearly in contact with the LPS (Figure 5A–F). Combining all of the data, these experimental and theoretical findings might indicate that all MSI-derived peptides can structurally adapt to the bacterial LPS, forming complexes that could be an initial step for endotoxin neutralization.

### Visualization of the Peptide’s Effect on LPS Aggregates

**Figure 6:**
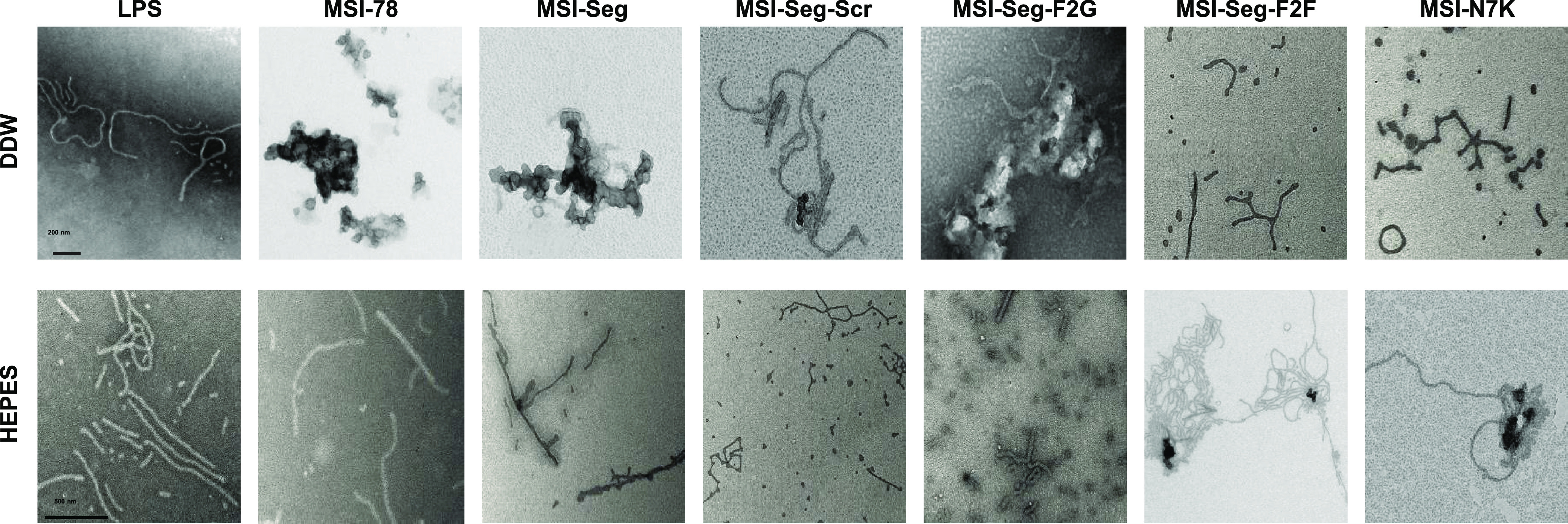
Transmission electron microscopy of all peptides in the presence of LPS dissolved in DDW and HEPES. LPS (10 μM) was dissolved in DDW and HEPES, and each peptide was added in a concentration of 1 μM (scale bar: 200 nm in DDW and 500 nm in HEPES).

LPS is known to have a well-defined structure when in solution, and it has been observed to aggregate beyond its critical micelle concentration (CMC). Electron microscopy can detect these aggregates as thick and mixed fibers. We investigated the effect of the peptides on the LPS aggregates, and we observed that MSI-78 presented more loose aggregates. A similar phenomenon was observed with the treatment of MSI-Seg. Treatment with MSI-Seg-F2G was smudge and amorphous, which correlates with its in vitro activity. MSI-Seg-Scr and MSI-N7K first present more thick fibers with a new structure of combined sticks. The same phenomenon was observed in the sample treated with MSI-Seg-F2F, in which we also see small segments and not long fibers. These results support the in vitro analysis (cytokine secretion). Peptides with the ability to neutralize LPS caused the LPS aggregates to adopt a more relaxed, thick, and open conformation. On the other hand, the inactive peptide formed undefined smear aggregates. (Figure 6).

### Confocal Imaging

**Figure 7:**
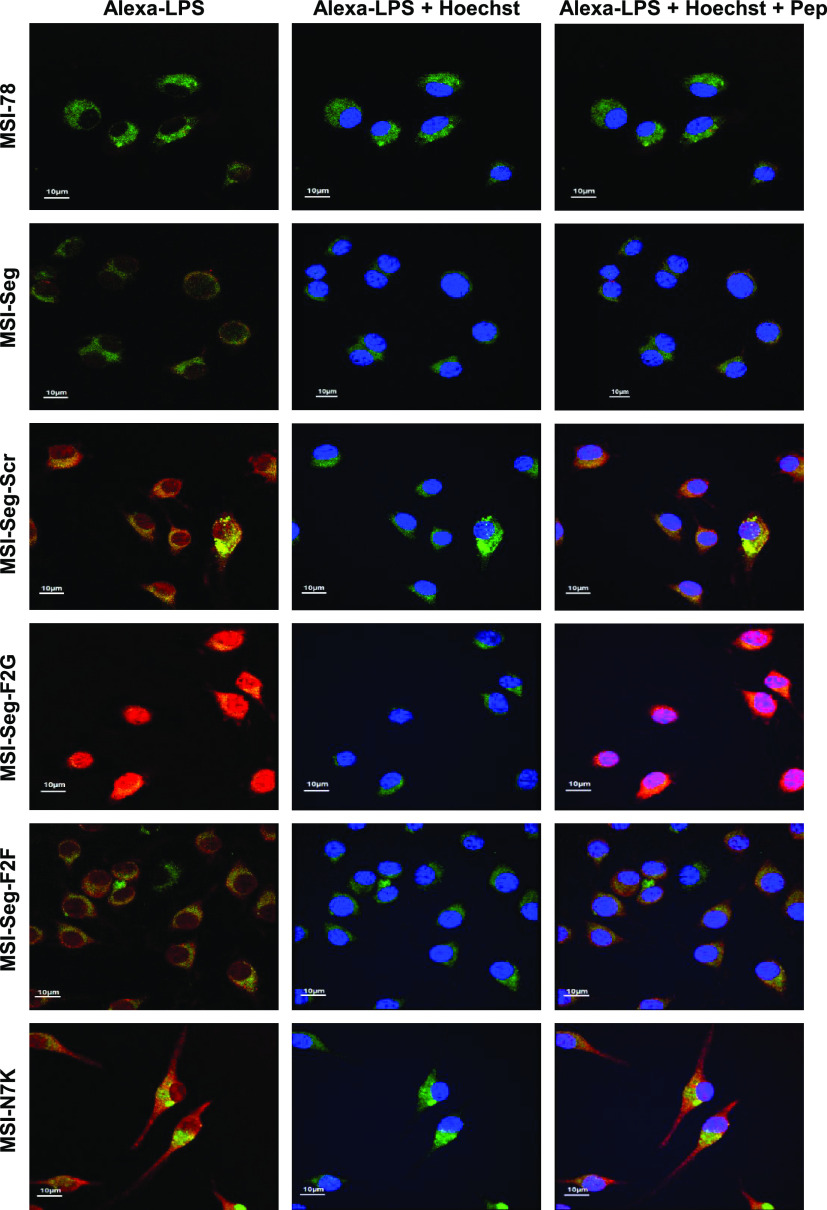
Confocal images of bone marrow-derived macrophages. A combination of the peptide and LPS-Alexa 488 was added to the cells for 1 min, then washed, and fixed with PFA 3.6%. The cells were examined for the peptides and LPS location in comparison to the membrane and nucleus (scale bar: 10 μm). Green color from Alexa Flour 288 for LPS labeling; blue color from Hoechst is used to stain DNA; red color from rhodamine is used to label the peptides.

To determine the distribution of a peptide between the nucleus and cytoplasm, confocal microscopy was employed. Bone marrow cells were collected and differentiated into macrophages. Following this, the cells were exposed to a combination of a peptide-TAMRA (red) and LPS-Alexa 488 (green) for 5 min, washed three times, and then fixed using PFA. Before imaging, Hoechst (1 μg mL^–1^) was added to the cells. Fiji was used to evaluate the percentage of a peptide located in the nucleus versus the cytoplasm. The values are presented between 0 and 1, 1 indicates that the labeling ratio between the nucleus and the cytoplasm is similar and 0 when the cytoplasm is more labeled. The data revealed a correlation between the labeling and the activities of the peptides. MSI-78 is directed close to the membrane. MSI-Seg-Scr and MSI-Seg-F2G are located in the cytoplasm and nucleus and are not as potent as the other peptides. MSI-Seg, MSI-Seg-F2F, and MSI-N7K show that similar values may be due to similar activities and structures. It is important to note that MSI-Seg and MSI-Seg-F2F share almost the same amino acid composition and organization (changes of two Phe from their L configration to their D) and have a similar penetration pattern (Figure 7).

### Image Flow Cytometry Analysis of MSI-Derived Peptides

Imaging flow cytometry (IFC) is a novel technique that combines two well-known techniques, flow cytometry and fluorescence microscopy, with high statistical analysis.^33,34^ By using IFC, we obtained detailed morphometric cellular analysis by simple labeling. It is known that charged peptides can easily enter the cell by different mechanisms using cell-penetrating peptides (CPPs).^35,36^ We used IFC to understand peptide penetration on RAW 264.7 cells in different conditions and times. We first examined the kinetics of entry to the cells. Cells were supplemented with different peptides and examined for 1 h (>10^5^ cells for each experiment). Internalization is the log scale of the ratio between the staining intensity inside and the total labeling, which was calculated and plotted against time (Figure S1A,B). Negative values indicate that the peptides are located close to the membrane (or around the membrane), while positive values indicated that the peptides are located inside the cells. The analysis revealed that, in MSI-78, internalization distribution was more limited and homogeneous, with a lower penetration value even after 1 h. Most of the peptide was located around the membrane, compared to the other peptides, which show similar internalization patterns with time between them.

**Figure 8:**
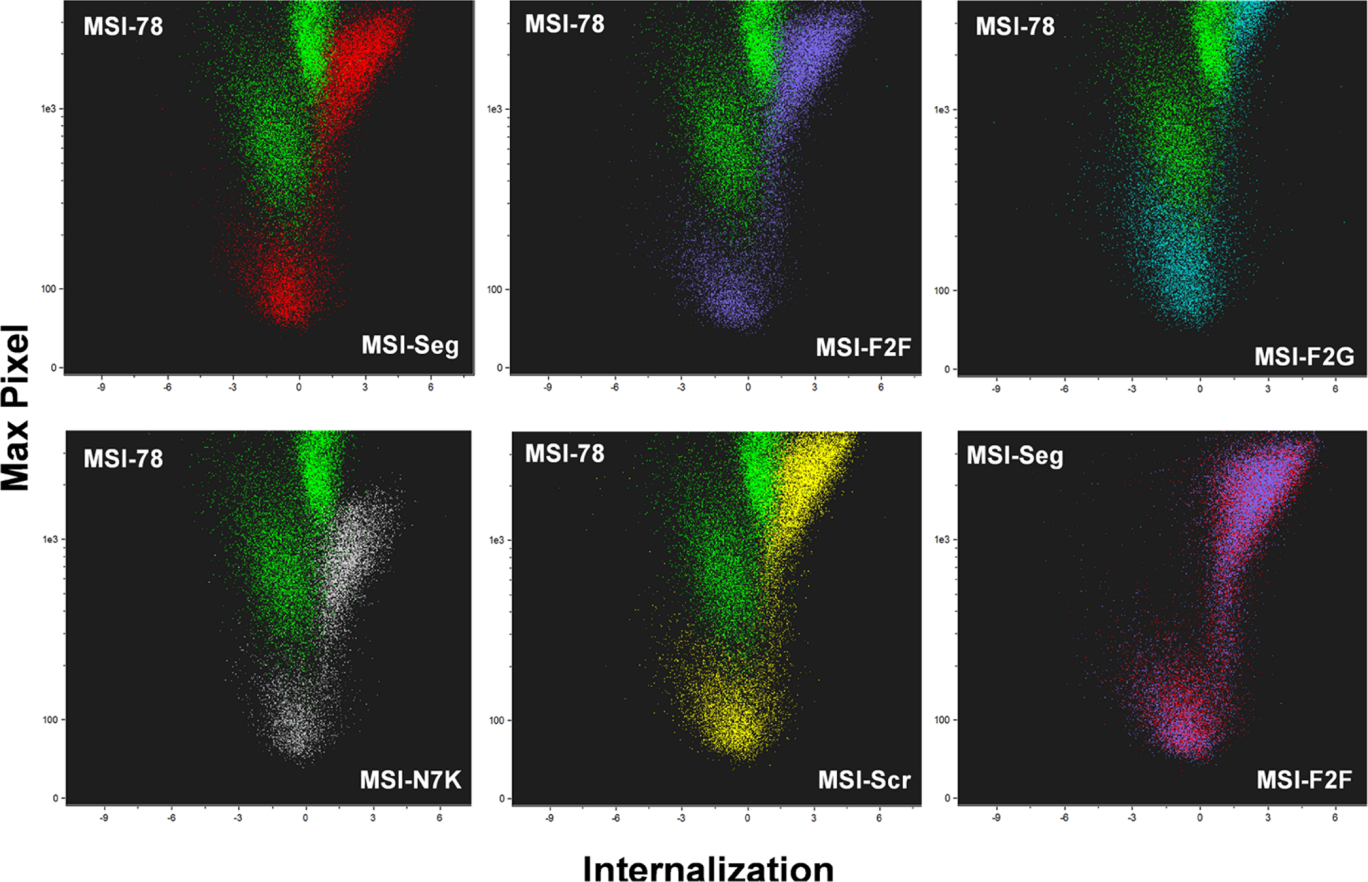
Comparison of the internalization and intensity values of the peptides in comparison to the MSI-78 which is presented in green (red is MSI-Seg, purple is MSI-Seg-F2F (MSI-F2F), light blue is MSI-Seg-F2G (MSI-F2G), gray is MSI-N7K, yellow is MSI-Seg-Scr (MSI-Scr)). Overlapping pattern of both MSI-Seg and MSI-Seg-F2F (MSI-F2F).

As time did not indicate a specific behavior, we tried to further separate the results into subpopulations. An analysis of cellular distribution vs the Max Pixel (the highest intensity pixel value within the image) was performed (Figure 8). This analysis demonstrated that the cells were divided into two populations without overlapping. MSI-78 had a different pattern with lower internalization values, which indicate its presence on the membrane environment, while the other showed higher values (inside the cell). MSI-Seg and MSI-Seg-F2F have the same amino acid composition, besides the two Phe having a D-isomer conformation (Figure 8).

## Discussion

Antimicrobial peptides have the potential to be developed as therapeutic agents against bacterial infections and may serve as candidates for novel antibiotics. The majority of existing treatments are focused on targeting accessory proteins, namely, LBP, CD14, and MD-2, which facilitate the activation of TLR4. LPS, known to cause an inflammatory response, activates the immune system via TLR4 and is a common feature in many inflammatory diseases, such as cancer, inflammatory bowel disease (IBD), diabetes and others, and sepsis.^13,37−42^ Until today, there is no effective therapy for sepsis, so it is urgent to develop antagonists based on the LPS structure to treat this.

Our study has demonstrated that, in addition to their antibacterial activity, antimicrobial peptides can bind to LPS and hinder its ability to trigger the immune system. This suggests that they could be considered as potential treatments for sepsis.^6,14,43^ We generated five analogues of MSI-78 by sequence alteration to study the effect on the antimicrobial and antiendotoxin activities. From MIC experiments, the data revealed that all of the peptides showed MICs 2-fold higher than the parental peptide MSI-78 except MSI-Seg-F2G, which might be due to hydrophobicity and positive charges of the amino acids rather than their specific sequence or structure. However, the data revealed that MSI-78 is more toxic toward RAW 264.7 and RBCs than its tested analogues. This toxicity reduction may be due to the sequence segregation of MSI-78, which lead to decreasing toxicity.

The impact of cationic and amphiphilic molecules on the aggregate structure of LPS has been extensively studied with regard to peptides and polymers.^31,40,41^ Activation of cells by LPS has indicated that AMPs interact with LPS, leading to modifications in its structure and physical properties, ultimately diminishing the bioactivity of this endotoxin. Cytokines are known markers of an inflammatory response. After treatment with peptides, the amount of proinflammatory cytokines secreted from RAW 264.7 cells, such as TNFα, IL-6, and NO, was reduced. MSI-Seg-F2F and MSI-N7K, the most active peptides significantly reduced cytokine levels at low concentrations. The reason behind the enhanced cytokine inhibitions observed in segregated peptides such as MSI-Seg-F2F and MSI-N7K may be attributed to the variation in their conformation and the interactions between oligosaccharides with different chirality, which are related to chirality. Further, the mode of action was studied by using various biophysical and biochemical methods. ATR-FTIR spectroscopy was used to determine the secondary structure of the peptides in the presence of LPC and LPS. The data revealed that MSI-78 displayed an α helical structure and MSI-N7K adopted a random coil structure, while all of the segregated peptides adopted β-sheet structure conformations in the LPS environment. Molecular dynamics simulations support their conformation and demonstrate a possible arrangement of the peptides when bound to LPS. All of the segregated peptides formed hydrophobic patches toward the lipid A moiety of LPS. Additionally, the electrostatic interactions are fundamental, directing the peptides to the negatively charged phosphate group of LPS. Furthermore, the MST study demonstrated that all peptides bind LPS at the same *K*_d_ values except MSI-Seg-F2F and MSI-N7K. Moreover, it is evident from microscopy that the active peptides detach LPS aggregates and form thinner structures, whereas the nonactive peptides form firm aggregates. Insights into the specific conformational requirements of LPS can be gained from studying the three-dimensional (3D) structures, interactions, and activities of MSI-Seg-F2F and MSI-N7K in relation to LPS.

## Conclusions

In conclusion, various biochemical and biophysical methods, including ATR-FTIR spectroscopy, MST and microscopic experiments, as well as all-atom MD simulations, were utilized to investigate the structural modifications of peptides when interacting with LPS. Based on the results of LPS neutralization and 3D modeling, it is hypothesized that the effectiveness of peptides is attributed to electrostatic interactions and hydrophobicity that direct them towards LPS molecules. The findings suggest that MSI-Seg-F2F and MSI-N7K have the ability to counteract the effects of endotoxins and may be a promising option for preventing sepsis. Furthermore, the new peptides exhibit lower levels of toxicity and demonstrate a wide range of efficacies, making them potentially valuable candidates for the development of treatments for severe infectious inflammation.

## Experimental Procedures

### Materials

Rink amide MBHA resin, 4-methyl-benzhydryl amine resin (MBHA), and 9 fluorenyl methoxycarbonyl (Fmoc) amino acids were obtained from Calbiochem-Novabiochem. Trifluoroacetic acid (TFA) (Sigma), piperidine (Merck), *N*,*N*-diisopropylethylamine (DIEA) (Sigma), *N*-methyl morpholine (NMM) (Fluka), *N*-hydroxy benzotriazole hydrate (HOBT) (Aldrich), 2-(1*H*-benzotriazole-1-yl)-1,1,3,3-tetramethyluronium hexafluorophosphate (HBTU), and dimethyl formamide (DMF, peptide synthesis grade) (Biolab) reagents were used for peptide synthesis. *E. coli* O111:B4 LPS and FITC-LPS were purchased from Sigma. Lipopolysaccharide from *E. coli* (055:B5) conjugated to Alexa 488 was purchased from Thermo Fisher Scientific. Tissue culture medium, serum, antibiotics, and supplements were purchased from Biological Industries (Beit Haemek, Israel).

### Peptide Synthesis and Purification

Peptides were produced via Fmoc solid phase synthesis on rink amide resin using an automated peptide synthesizer (Liberty Blue Automated MW Peptide Synthesizer 240v, ISI, Israel Scientific Instruments Ltd.). To label the peptide’s N-terminus with fluorescence, rhodamine-*N*-hydroxysuccinimide was dissolved in anhydrous dimethyl formamide (DMF) containing 2% *N*,*N*-diisopropylethylamine and applied to resin-bound peptides. The N-terminal Fmoc protecting group was removed by incubation with 20% piperidine for 10 min, while other reactive amine groups were protected. The resin-bound peptide was thoroughly washed with DMF and dichloromethane (DCM), dried, and cleaved by adding 95% trifluoroacetic acid, 2.5% H_2_O, and 2.5% triethylsilane for 3 h. The crude peptides were purified (>98% homogeneity) via reverse-phase high-performance liquid chromatography (RP-HPLC) using a C18 column (Grace Discovery Sciences, Deerfield, IL) and a linear gradient (10–90%) of acetonitrile (ACN) in water (both containing 0.1% TFA (v/v)) over 40 min. All peptides analyzed for biological activity exhibited a purity greater than 95% (Figures S3–S8).

### Antibacterial Activity

The peptide’s minimal inhibitory concentration (MIC) was examined as previously described.^44^ In brief, sterile 96-well U-bottom polystyrene plates were used to evaluate peptide activity. Bacterial cells including *S. Typhi* ATCC 14028s, *E. coli* ATCC 35218, *S. aureus* ATCC P8538, and *Lactobacilli* ATCC 25258 were cultured in Mueller Hinton broth (MHB) at 37 °C overnight. The cells were then washed, centrifuged, and re-suspended in MHB medium. Afterward, 50 μL aliquots of suspended bacteria (1 × 10^6^ colony forming units, CFU mL^–1^) were added to 50 μL BM2 medium, which contained peptides in serial twofold dilutions. The plates were then incubated at 37 °C for 18 h. The inhibition of growth was determined by measuring the absorbance at 600 nm using a microplate autoreader. The MIC was defined as the concentration at which 90% growth inhibition was observed after 18 h of incubation.

### Cell Culture

The majority of the in vitro assays were conducted using RAW 264.7 murine macrophages (ATCC-TIB71) that were cultured in DMEM supplemented with 10% FBS (low endotoxin), l-glutamine, sodium pyruvate, nonessential amino acids, and antibiotics (Biological Industries, Beit Haemek, Israel). The cells were maintained in an incubator at 37 °C under a humidified atmosphere containing 5% CO_2_.

### Bone Marrow-Derived Macrophages

Bone marrow cells from femurs and tibiae were gathered and cultured in an RPMI medium that included 10% FBS, 1% l-glutamine, and 10 ng mL^–1^ MCSF-1 recombinant (Peprotech). This growth factor is specific to the lineage and induces the cells to differentiate into macrophages.^45^ After 3 days, half of the medium was renewed, and on the 7th day, the cells were utilized for a fluorescent labeling assay and visualized using an Olympus FV1000 confocal microscope [60× objective lens (oil)]. Further, Fiji was used for image analysis.^46^

### LD_50_ Calculation

LD_50_ is the calculated amount of chemical that causes the death of 50% of the population tested, in this case, RAW 264.7. Each well was seeded with 1 × 10^5^ cells 1 day before the experiment. Peptides were added in several doses and incubated with the cells for 2 h, followed by 2 h of XTT. We performed a linear graph and calculated the concentration in which 50% were dead.

### XTT Cytotoxicity Assays

DMEM supplemented with low endotoxin FBS (10%), l-glutamine (1%), sodium pyruvate (1%), pen–strep (1%), and unessential amino acids (1%) was used to grow RAW 264.7 cells at 37 °C in a humidified atmosphere containing 5% CO_2_ and 95% air. A 96-well Falcon plate was utilized for the assay, with each well containing 1 × 10^5^ cells in a 200 μL medium, cultured overnight and washed with phosphate-buffered saline (PBS) before the experiment. Peptides were added to the wells at various concentrations for 2 h, followed by XTT with fresh medium for another 2 h. The optical density was measured at 450 nm using an enzyme-linked immunosorbent assay plate reader, and cell viability was determined relative to the control.

### Hemolytic Assay

To examine the hemolytic properties of the peptides, a concentration of 100 μM was used. A 4% suspension of pig erythrocytes, freshly collected and washed three times in phosphate-buffered saline (PBS, pH 7.3), was used for this purpose. After incubation at 37 °C for 1 h, the suspension was centrifuged at 800*g* for 10 min, and the absorbance at 540 nm was measured. Complete hemolysis was determined by adding 1% Triton X-100, while adding only PBS was used to determine 0% hemolysis.The hemolysis calculation was as follows: % hemolysis = (OD_Peptide_ – OD_Buffer_)/(OD_Triton1%_ – OD_Buffer_) × 100.

### Neutralization of Endotoxins

In a 96-well plate, 1 × 10^5^ cells were cultured overnight. The next day, the cells were provided fresh DMEM medium containing all necessary supplements. Peptides, dissolved in DMSO, were added simultaneously with the LPS (10 ng mL^–1^) and LTA analogue Pam3CSK4 (100 ng mL^–1^) at various concentrations. The final concentration of DMSO was 1%, and the water concentration was 0.5% for all groups. Cells were incubated for 4 h, and then media samples from each treatment were collected and stored at −20 °C. The concentration of TNF-α in each sample was assessed using a mouse TNF-α enzyme-linked immunosorbent assay kit (Biosource ELISA, Invitrogen) following the manufacturer’s instructions. All experiments were conducted in triplicate.

### Transmission Electron Microscopy (TEM)

Using a FEI Tecnai T12 TEM electron microscope operating at 120 kV, samples were examined. A 10 μL mixture of peptide and LPS at the same molar ratio was deposited on a 400 mesh copper grid coated with a carbon-stabilized formvar film. After 1 min, excess fluid was eliminated, and the sample was negatively stained with 2% uranyl acetate dissolved in water. After 1 min, the excess uranyl was removed from the grid, and the samples were observed.

### Attenuated Total Reflection Fourier Transform Infrared (ATR-FTIR) Spectroscopy

A Nicole 380 (Thermo Electron Corp.) was used to obtain the spectra. Peptides were measured at concentrations of 3–5 mg mL^–1^ in the presence of 3 mg mL^–1^ LPS or 10 mg mL^–1^ LPC, which mimics the membrane environment. The solvent spectrum was subtracted, and the baseline corrected spectra were processed. The Voigt profile bands in the amide(I) region (1700–1550 cm^–1^) were fitted using PEAKFIT (Jandel Scientific) software. A nonlinear, least-squares optimization method was used to optimize the bands until the experimental spectrum had an *r*^2^ (least-squares factor) > 0.996. The secondary structure was estimated by comparing the area of individual peaks representing different secondary structural elements to the area of the amide(I) band.

### Molecular Modeling

At first, attempts were made to create 3D models of wild-type peptide MSI-78 and its analogues through homology modeling simulations. However, due to the absence of significant template structures, the ab initio server QUARK^47^ was utilized to generate theoretical models in 3D. These models were ranked based on their free-energy values. The quality of the models with the lowest free-energy values was then evaluated using the ProSa server by comparing their overall quality scores with those obtained for proteins resolved by X-ray crystallography or nuclear magnetic resonance (NMR) approaches.^32^ PROCHECK^48^ assessed the geometry, stereochemistry, and energy distribution of the peptide by calculating its average score for dihedral angles and covalent forces. Finally, to produce the KKKKKGIGDFLADFGAFVILKKKK-NH2 peptide, the chirality of the Cα atoms of Phe 9 and Phe 12 was modified using the Maestro v. 10.2.011 Schrodinger.

### Molecular Docking

To understand the atomic interactions between the crystal structure of lipopolysaccharide (LPS) and the lowest free-energy theoretical models resulting from molecular modeling simulations, molecular docking studies were conducted. A grid box with a spacing of 1 Å and dimensions of 40 × 40 x 40 points was constructed on AutoDock Tools.^49,50^ Peptide/LPS pairs were placed in the grid box, with nonpolar hydrogens added and maximum freedom for peptide side chains unlocked. Using AUTODOCK v. 4.2,^49,50^ 50 molecular docking simulations were performed, and the resulting peptide/LPS complexes were arranged based on their affinities in kcal mol^–1^. To visualize and measure atomic interactions, PyMOL was used, with a maximum distance of 3.6 Å between atoms being respected.

### Microscale Thermophoresis

MST analysis was performed to analyze the binding between MSI-derived peptides and LPS labeled with FITC using a NanoTemper Monolith NT.115 apparatus (NanoTemper Technologies, Germany).^51^ To assess for any nonspecific binding, an initial capillary scan was performed. This involved dissolving a high concentration of each peptide in PBS and adding it to LPS-FITC. The peptides were then titrated in 1:1 dilution, starting from a concentration of 1 mM. Binding constant (*K*_d_) values were determined using MST analysis.

### Image Stream Analysis

To test how each of the peptides behaves in solution, we labeled all of the peptides with rhodamine, while LPS was labeled with Alexa 488. Bone marrow-derived macrophages were treated with both peptide and LPS for 1, 15, and 30 min. Then, the cells were fixed with 3% paraformaldehyde (PFA) for 15 min at RT, followed by three washes with PBS. To check only the live cells, we labeled their nucleus with Hoechst (1 μg mL^–1^). Cells were then analyzed using an imaging flow cytometer, ImageStreamX mark II (Amnis, Part of Luminex, Au. TX). Lasers used were 405 nm (120 mW), 488 nm (100 mW), 561 nm (30 mW), and 785 (5 mW). At least 5 × 10^4^ cells were collected from each sample. Images were analyzed using IDEAS 6.2 software (Amnis, Part of Luminex, Au. TX). Cells were gated according to their DNA content using the area (in square microns) vs the intensity (arbitrary units) of the Hoechst staining (channel 7). Single cells were then gated according to the area vs aspect ratio intensity (the ratio between the minor axis and the major axis of a best-fit ellipse for the nuclear object, intensity weighted) of the Hoechst staining. To include only cells that were in focus, the contrast and gradient RMS features (measures the sharpness quality of an image by detecting large changes of pixel values) of the bright-field image were used. Cropped cells were eliminated by plotting the area of the bright-field image vs the Centroid X feature (the number of pixels in the horizontal axis from the upper left corner of the image to the center of the mask). Cells positively labeled for the peptide were gated according to the intensity (total amount of fluorescence within the image) vs Max Pixel (the value of the highest intensity pixel) of the rhodamine staining (channel 4). To quantify the internalization of the peptide, first, a mask was created based on the bright-field image to contain the cytoplasm without the cell membrane (AdaptiveErode (M01, BF,70)). Then, the internalization feature was calculated upon this mask (the ratio of the intensity inside the cell to the intensity of the entire cell, mapped to a log scale). The higher the score, the greater the concentration of intensity inside the cell. Internalized cells typically have positive scores, while cells with little internalization have negative scores. Cells with scores around 0 have a mix of internalization and membrane intensity.

### Statistical Analysis

Statistical significance was determined using one-way ANOVA (*p ≤ 0.05, **p ≤ 0.01, and ***p ≤ 0.001) by Prism. The results are shown as means ± standard errors of the mean unless indicated otherwise.

## Supporting Information Available

Internalization of peptides; in silico interaction between antimicrobial peptides and LPS; and effect of the peptides on TNF-α secretion by macrophages stimulated with LTA and HPLC chromatograms (PDF)
