# Supplementary material for: Interaction of Pexiganan (MSI-78)-Derived Analogues Reduces Inflammation and TLR4-Mediated Cytokine Secretion: A Comparative Study
Source: ACS Omega. 2023 May 12;8(20):17856–68. doi: 10.1021/acsomega.3c00850 (PMC10210221; doi:10.1021/acsomega.3c00850)
Supplement: Supplementary file 1 — ao3c00850_si_001.pdf [file ao3c00850_si_001.pdf]

Supplementary Information

Interaction of Pexiganan (MSI-78) Derived Analogs Reduce Inflammation and TLR4 mediated Cytokine Secretion: A Comparative Study

Hadar Cohen[a, ‡], Naiem Ahmad Wani[a, ‡], Daniel Ben Hur[a], Ludovico Migliolo[b], Marlon H. Cardoso[e,f,g], Ziv Porat[c], Eyal Shimoni[d], Octavio Luiz Franco[b,e,f], Yechiel Shai[a]*

[a]Department of Biomolecular Sciences, The Weizmann Institute of Science, Rehovot 76100, Israel

[b]Departamento de Engenharia Sanitária e Ambiental, Universidade Católica Dom Bosco, Campo Grande, 79117-900, Brazil

[c]The Department of Life sciences Core Facilities, The Weizmann Institute of Science, Rehovot 76100, Israel

[d]Department of Chemical Research Support, The Weizmann Institute of Science, Rehovot 76100, Israel

[e]S-inova, Programa de Pós-Graduação em Biotecnologia, Universidade Católica Dom Bosco, Campo Grande-MS 79117900, Brazil;

[f]Centro de Análises Proteômicas e Bioquímicas, Pós-Graduação em Ciências Genômicas e Biotecnologia, Universidade Católica de Brasília, Brasília-DF 70790160, Brazil;

[g]Instituto de Biociências (INBIO), Universidade Federal de Mato Grosso do Sul, Cidade Universitária, 79070900 Campo Grande, Mato Grosso do Sul, Brazil

*To whom correspondence should be addressed, at the Department of Biomolecular Sciences,

The Weizmann Institute of Science, Rehovot, 76100 Israel.

Tel: 972-8-9342711; Fax: 972-8-9344112

E-mail: Yechiel.Shai@weizmann.ac.il

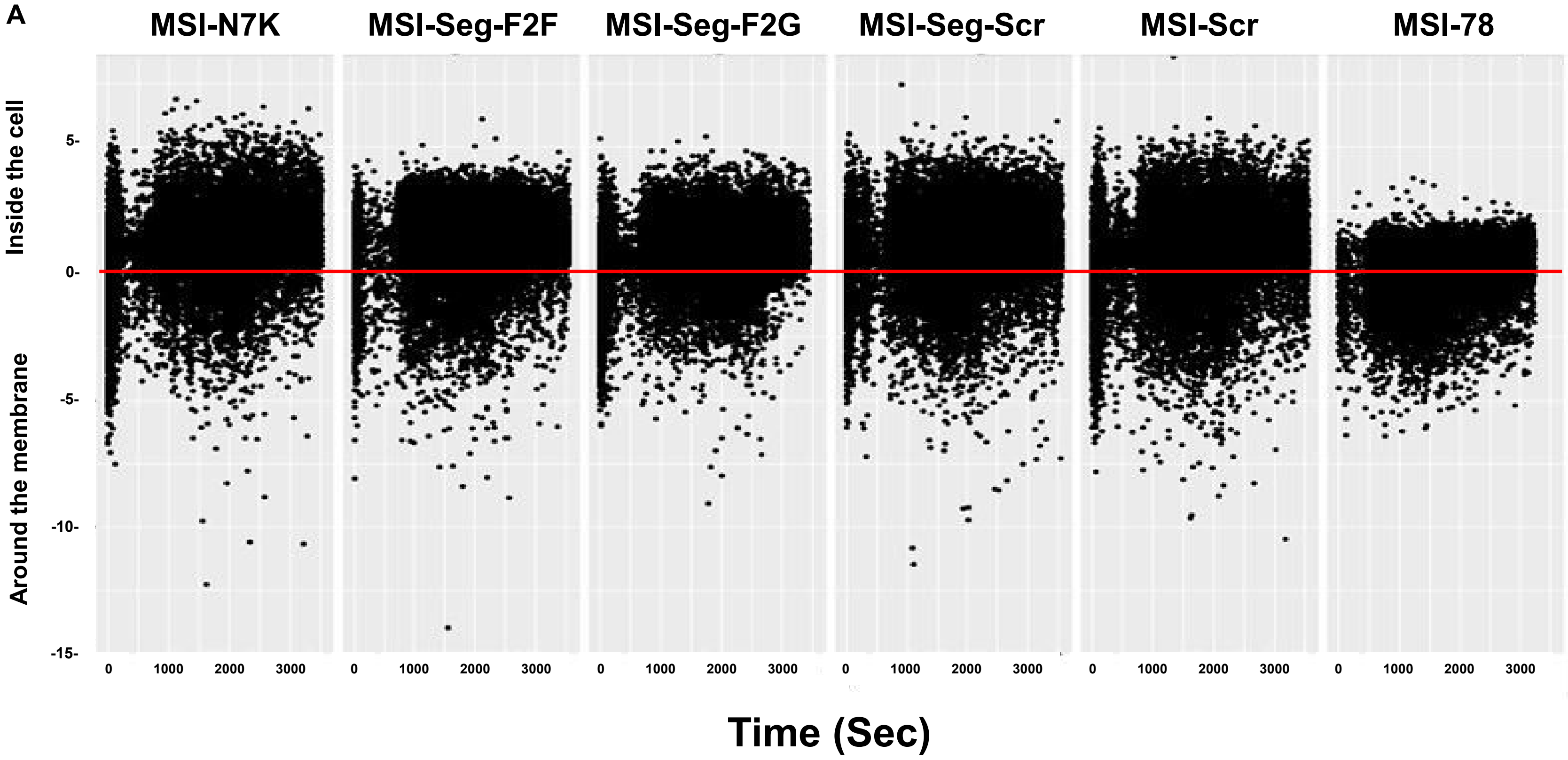

A
MSI-N7K
MSI-Seg-F2F
MSI-Seg-F2G
MSI-Seg-Scr
MSI-Scr
MSI-78
Inside the cell
Around the membrane
5-
0-
-5-
-10-
-15-
0
1000
2000
3000
Time (Sec)

B

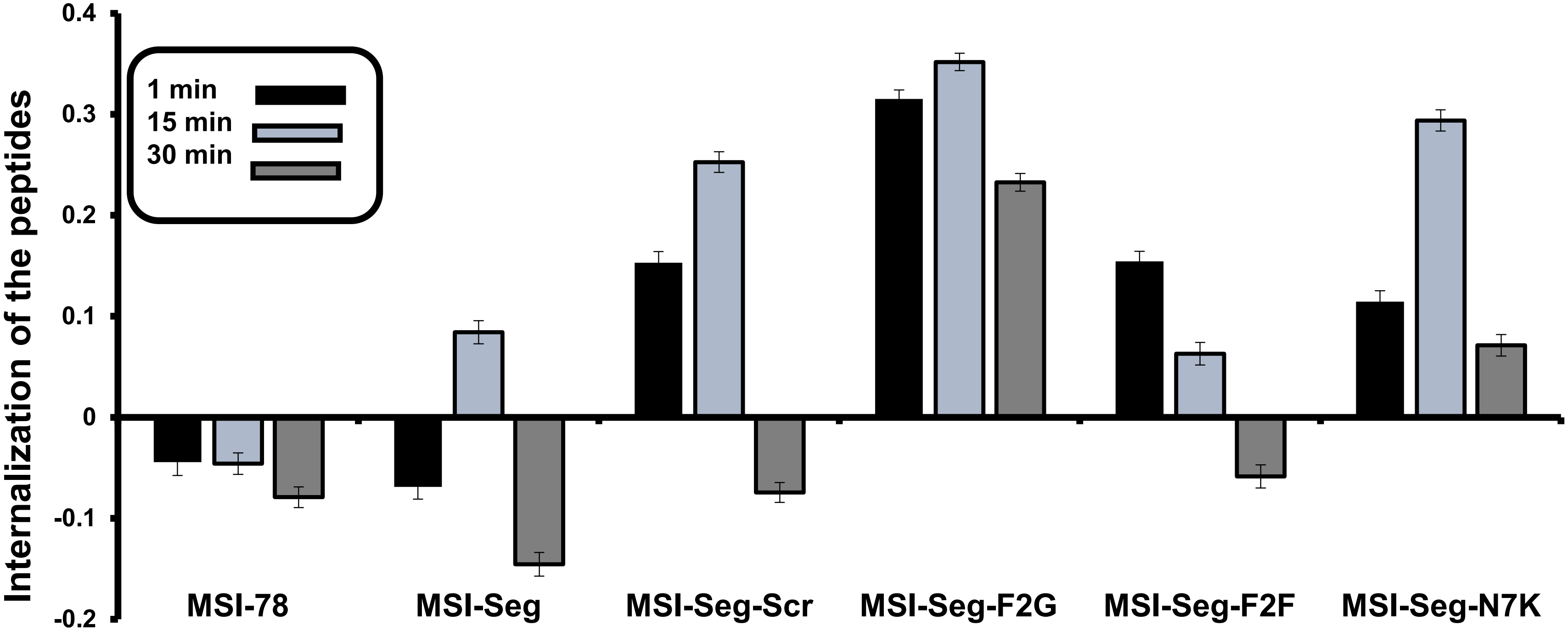

Figure S1. (**A**) Internalization values of all peptides during 60 min. The red line is the separation between the cells that are located on the membrane (negative values) and the ones that are inside the cell (positive values) (**B**) Membrane Image stream analysis i.e Internalization of each of the peptides towards RAW264.7 cells at different time points. More positive values indicates that the peptides are inside the cells while negative values represents outer/membrane location.

Table S1. *In silico* interaction between antimicrobial peptides (specify) and LPS.

MSI-F2F -4.4 kcal.mol^{-1}			Distances (Å)	LPS		Interactions
Residue	Position	Atom		Group	Atom	
Lys	1	NZ	3.2	Acyl chain	O2	SB
Lys	2	NZ	3.2	Phosphate	O4	SB
Lys	4	NZ	3.0	Acyl chain	O3	SB
Lys	5	NZ	3.2	Glucosamine	O4	SB
Phe	6	O3	3.3	Glucosamine	O4	HB
Phe	15	CZ	3.6	Acyl chain	C4	H
Lys	20	NZ	3.3	Kdo	O1B	SB
Lys	22	NZ	3.1	Phosphate	O2	SB
Lys	22	NZ	3.0	Kdo	O4	SB
MSI-N7K -4.1 kcal.mol^{-1}				LPS		
Lys	2	NZ	2.8	Kdo	O8	SB
Lys	3	NZ	3.3	Phosphate	O2	SB
Lys	4	O	3.0	Kdo	O1B	HB
Lys	5	NZ	3.2	Glucosamine	N2	HB
Lys	6	NZ	2.9	Phosphate	O2	SB
Lys	7	NZ	2.9	Acyl chain	O3	SB
Phe	14	CE2	3.6	Acyl chain	C7	H
Val	18	CG2	3.6	Acyl chain	C12	H
MSI-78 -4.0 kcal.mol^{-1}				LPS		
Lys	4	NZ	3.1	Phosphate	O2	SB
Lys	7	NZ	3.1	Acyl chain	O2	SB

Lys	10	NZ	3.3	Kdo	O8	SB
Phe	12	CE2	3.4	Acyl chain	CZ	H
Lys	14	NZ	3.2	Kdo	O5	SB
Ala	15	O	3.1	Acyl chain	O3	HB
Lys	18	NZ	2.9	Acyl chain	O2	SB
Lys	18	NZ	3.0	Acyl chain	O3	SB
**MSI-F2G -4.2 kcal.mol^{-1}**				**LPS**		
Lys	1	NZ	3.2	Phosphate	O2	SB
Lys	1	N	3.3	Acyl chain	O3	HB
Lys	4	NZ	3.0	Kdo	O8	SB
Lys	4	N	3.3	Kdo	O8	HB
Phe	15	CZ	3.3	Acyl chain	C9	H
Lys	19	NZ	3.0	Phosphate	O2	SB
Lys	21	NZ	2.9	Glucosamine	O7	SB
Lys	21	NZ	2.9	Kdo	O7	SB
Lys	21	NZ	3.1	Kdo	O8	SB
Lys	22	NZ	2.8	Phosphate	O2	SB
Lys	22	NZ	3.1	Kdo	O4	SB
**MSI-Scr -4.3 kcal.mol^{-1}**				**LPS**		
Lys	2	NZ	3.2	Glucosamine	O7	SB
Lys	2	NZ	3.0	Phosphate	O2	SB
Lys	2	NZ	3.3	Phosphate	O4	SB
Lys	4	NZ	3.0	Acyl chain	O3	SB
Lys	5	NZ	3.0	Kdo	O1A	SB
Lys	5	NZ	3.2	Glucosamine	O4	SB
Phe	6	O	3.1	Glucosamine	O4	HB
Gly	8	O	3.4	Acyl chain	O3	HB

Val	15	CG1	3.5	Acyl chain	C9	H
Ala	17	CB	3.1	Acyl chain	C8	H
Lys	20	NZ	2.8	Kdo	O6	SB
Lys	20	NZ	3.3	Kdo	OB1	SB
Lys	22	NZ	2.9	Kdo	O5	SB
Lys	22	NZ	3.3	Kdo	O4	SB
**MSI-Seg -4.1 kcal.mol^{-1}**				**LPS**		
Lys	3	NZ	3.0	Acyl chain	O3	SB
Phe	12	CE2	3.3	Acyl chain	C12	H
Lys	20	NZ	3.0	Kdo	O5	SB
Lys	20	O	2.8	Glucosamine	O4	HB
Lys	21	NZ	3.1	Acyl chain	O2	SB
Lys	21	NZ	3.3	Glucosamine	N2	HB
Lys	21	O	3.2	Phosphate	O2	HB
Lys	22	OXT	2.9	Glucosamine	O2	HB
Lys	22	NZ	3.2	Phosphate	O4	SB
Lys	22	NZ	3.4	Phosphate	O2	SB

Legends: HB: hydrogen bond; SB: saline bond; H: hydrophobic; NZ: positively charged Nitrogen atom of the side chain; Kdo: 3-deoxy-D-manno-2-octulosonic acid.

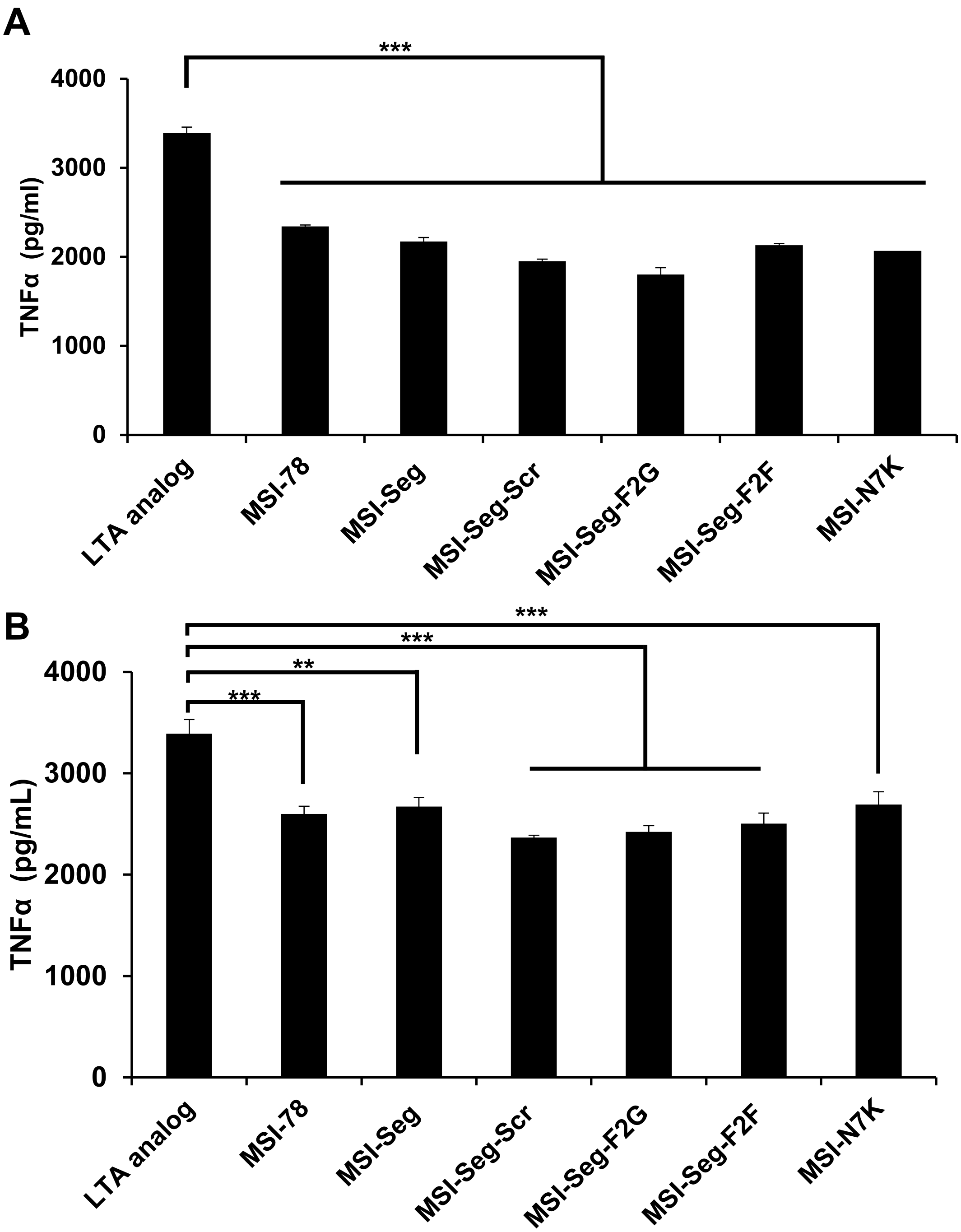

Figure S2: Effect of the peptides on TNF-α secretion by macrophages stimulated with LTA analog Pam3CSK4 (100 ng/mL) in the absence or presence of each of the different peptides at (A)1 μM and (B) 500 nM by the ELISA assay. Untreated cells served as the controls. Results are the mean ± SE of three independent experiments. One-way analysis of variance was used to analyze the data.

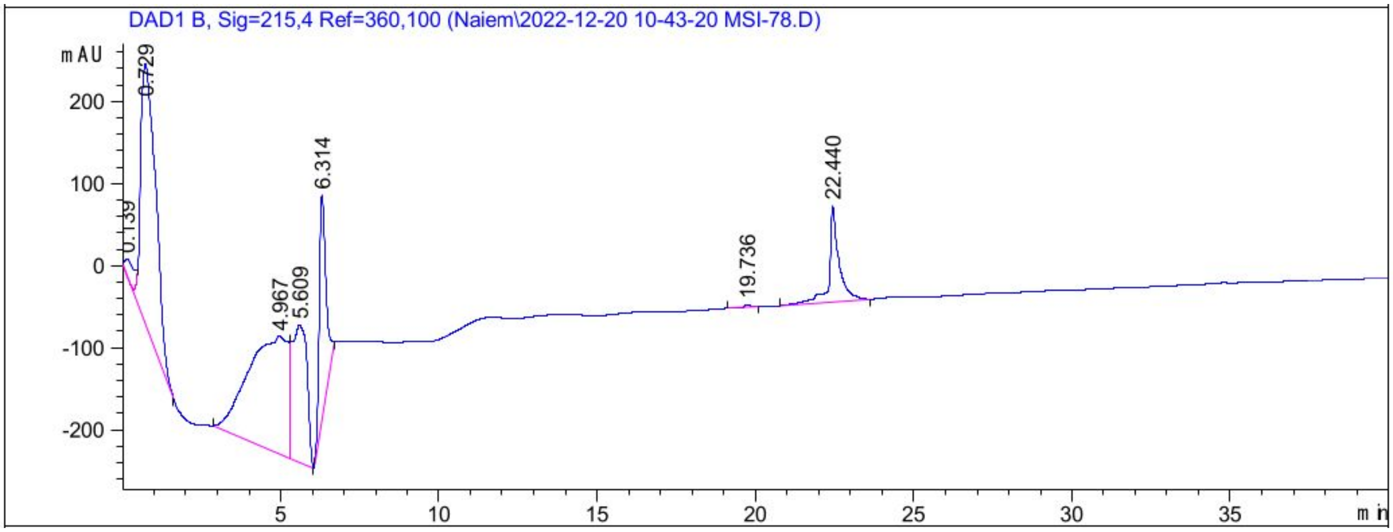

Figure S3: HPLC chromatogram of peptide MSI-78

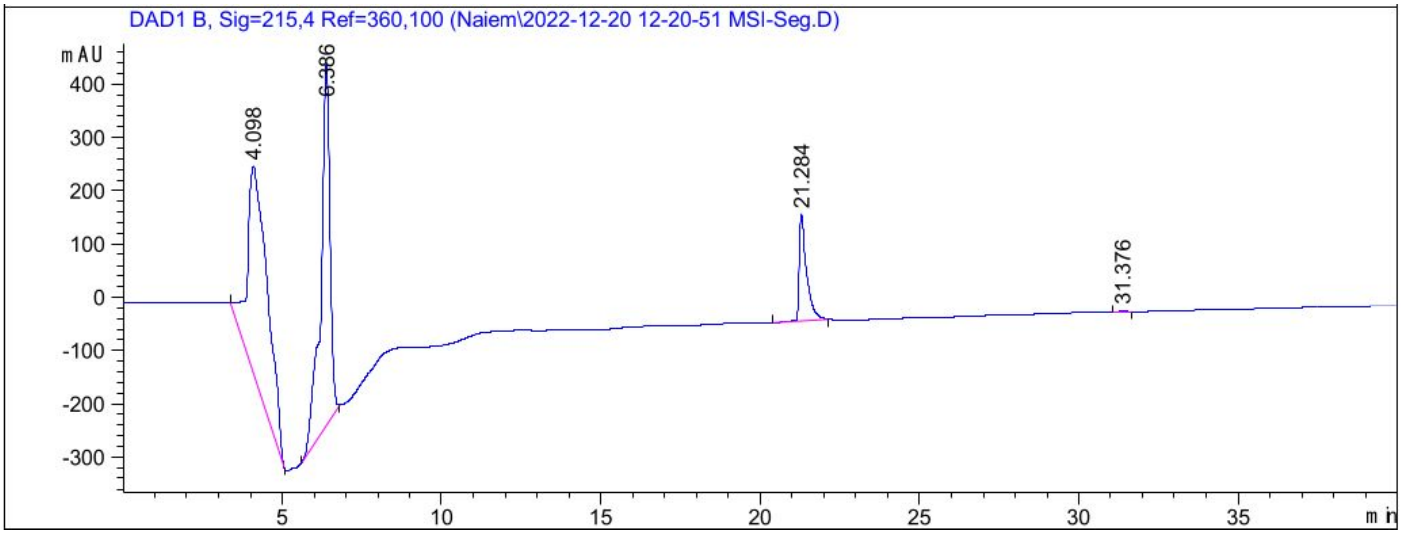

Figure S4: HPLC chromatogram of peptide MSI-Seg

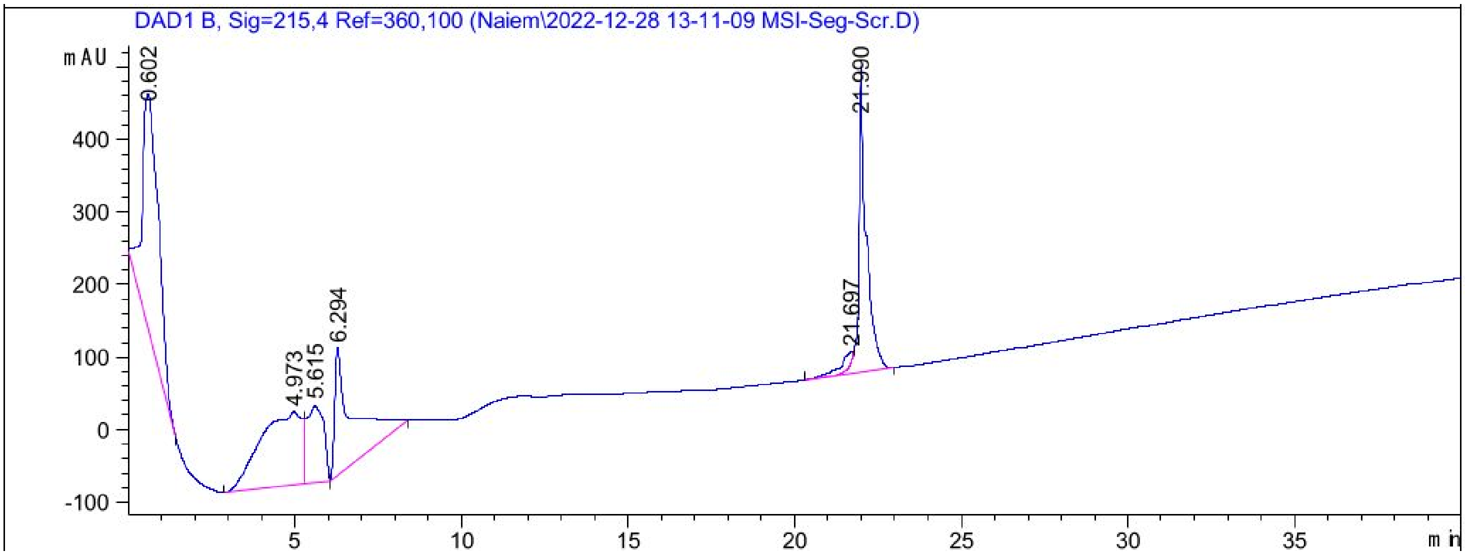

Figure S5: HPLC chromatogram of peptide MSI-Seg-Scr

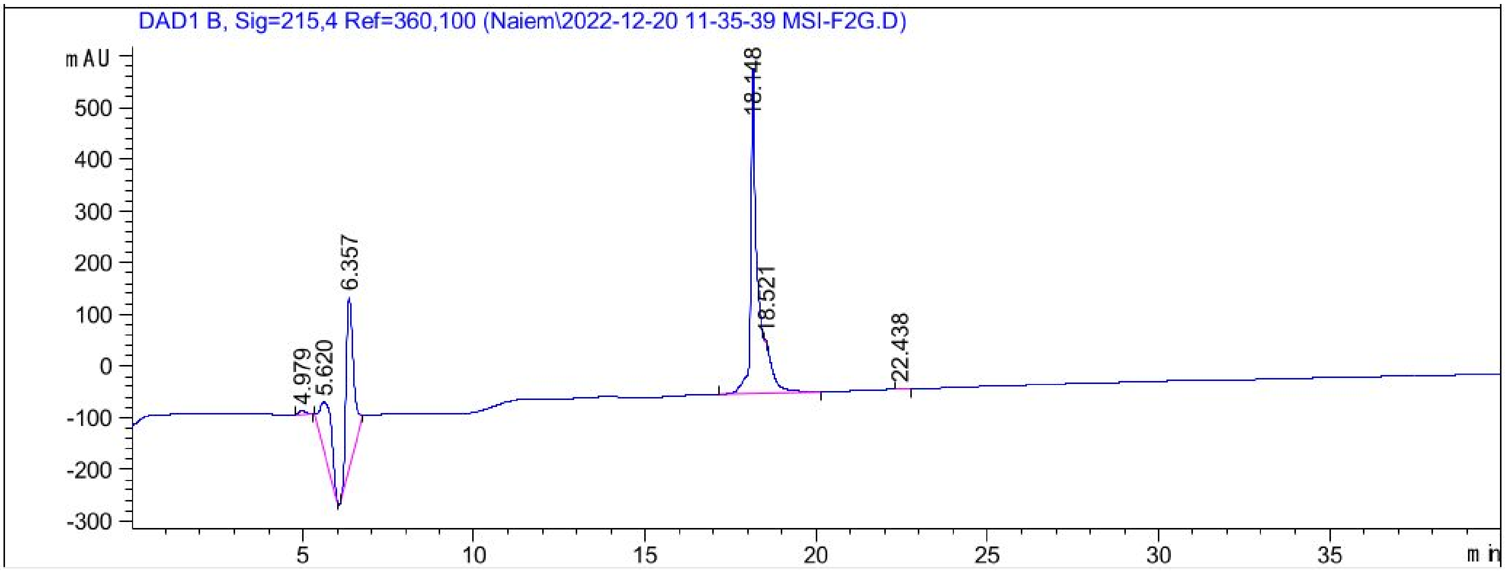

Figure S6: HPLC chromatogram of peptide MSI-F2G

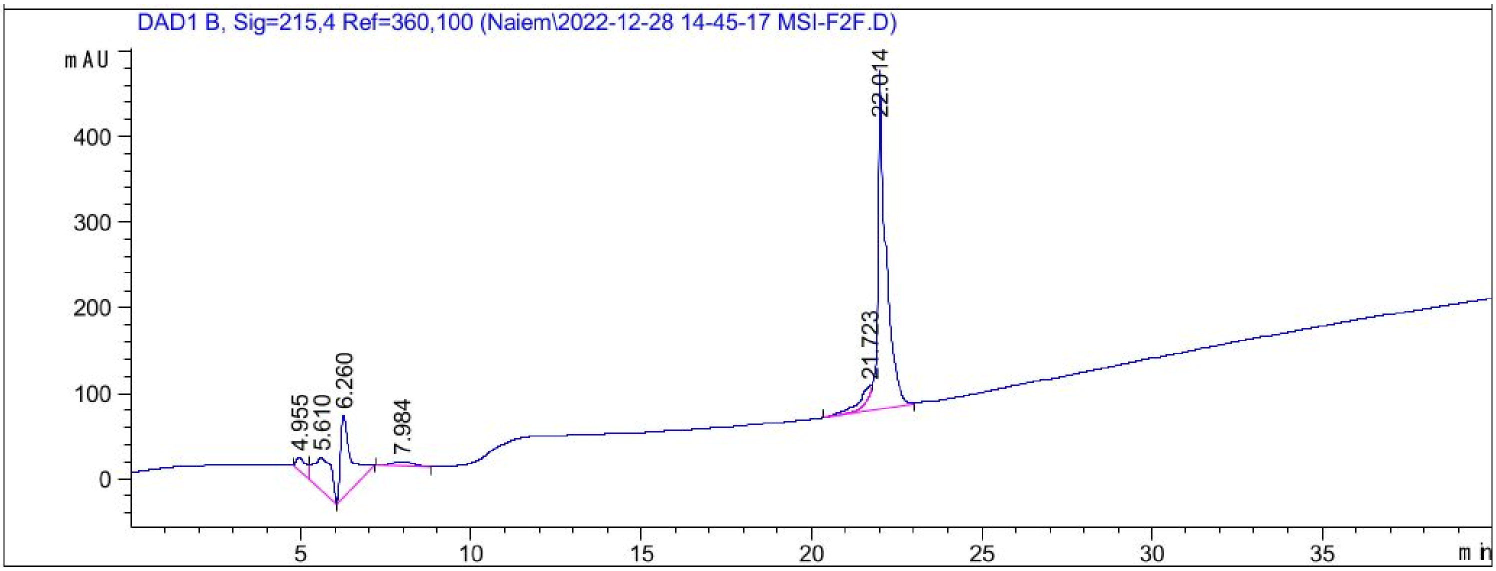

Figure S7: HPLC chromatogram of peptide MSI-F2F

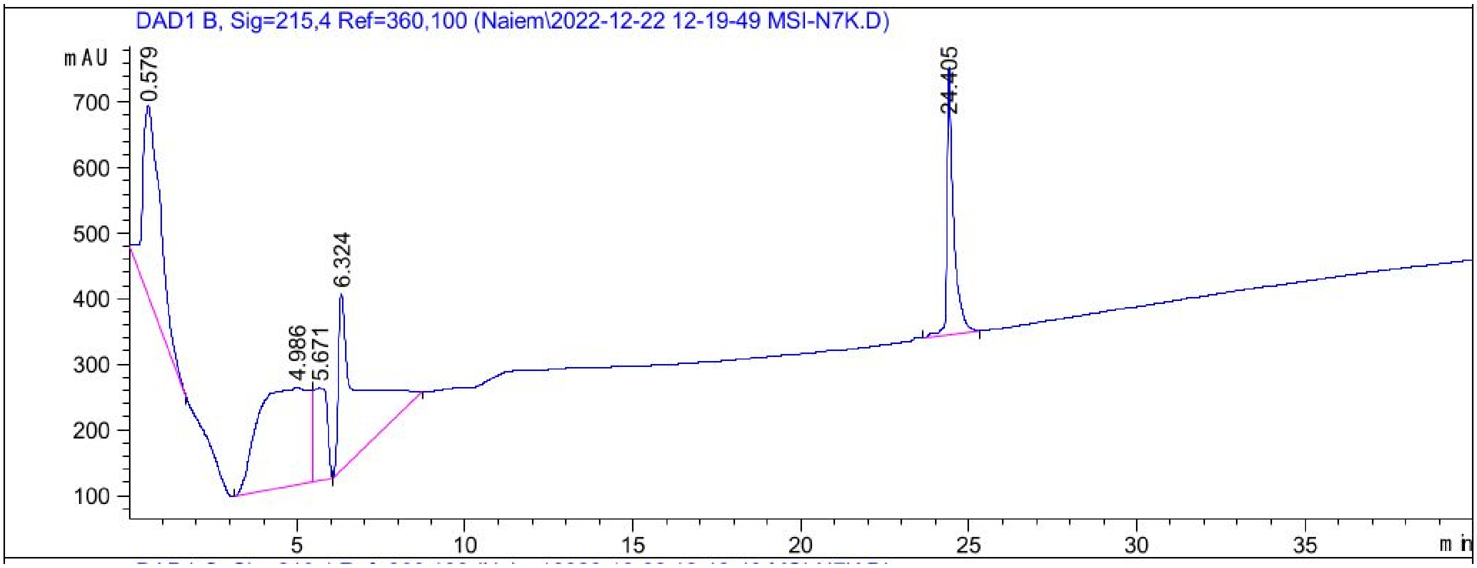

Figure S8: HPLC chromatogram of peptide MSI-N7K
